# Non-small cell lung cancer detection through knowledge distillation approach with teaching assistant

**DOI:** 10.1371/journal.pone.0306441

**Published:** 2024-11-06

**Authors:** Mahir Afser Pavel, Rafiul Islam, Shoyeb Bin Babor, Riaz Mehadi, Riasat Khan

**Affiliations:** Electrical and Computer Engineering, North South University, Dhaka, Bangladesh; The University of Texas, MD Anderson Cancer Center, UNITED STATES OF AMERICA

## Abstract

Non-small cell lung cancer (NSCLC) exhibits a comparatively slower rate of metastasis in contrast to small cell lung cancer, contributing to approximately 85% of the global patient population. In this work, leveraging CT scan images, we deploy a knowledge distillation technique within teaching assistant (TA) and student frameworks for NSCLC classification. We employed various deep learning models, CNN, VGG19, ResNet152v2, Swin, CCT, and ViT, and assigned roles as teacher, teaching assistant and student. Evaluation underscores exceptional model performance in performance metrics achieved via cost-sensitive learning and precise hyperparameter (alpha and temperature) fine-tuning, highlighting the model’s efficiency in lung cancer tumor prediction and classification. The applied TA (ResNet152) and student (CNN) models achieved 90.99% and 94.53% test accuracies, respectively, with optimal hyperparameters (alpha = 0.7 and temperature = 7). The implementation of the TA framework improves the overall performance of the student model. After obtaining Shapley values, explainable AI is applied with a partition explainer to check each class’s contribution, further enhancing the transparency of the implemented deep learning techniques. Finally, a web application designed to make it user-friendly and classify lung types in recently captured images. The execution of the three-stage knowledge distillation technique proved efficient with significantly reduced trainable parameters and training time applicable for memory-constrained edge devices.

## Introduction

The lungs function as crucial respiratory organs. Anatomically, the left lung offers increased internal space to the cardiovascular system. Inhalation, initiated by lung expansion, induces chest elevation, while exhalation involves lung contraction. The pivotal role of the lungs lies in oxygenating the circulatory system, where blood, laden with carbon dioxide and deficient in oxygen, undergoes purification during its journey from the heart. The lungs absorb oxygen and expel carbon dioxide, which disperses upon exhalation. The path of oxygen entails traversal through the esophagus, larynx, trachea, and bronchi before reaching the alveoli. These capillary-filled alveoli aid in the exchange of carbon dioxide for oxygen. Human respiration, essential for sustaining life, is a continuous process facilitated by the lungs supplying the bloodstream with vital air [1]. Cancer, a complex affliction, can simultaneously manifest in various forms across multiple organs.

Lung cancer is a significant concern in the world [2]. Non-small cell lung cancer (NSCLC) seems more aggressive than small-cell lung cancer. Lung cancer mainly occurs in two primary types: Small cell lung cancer (SCLC) and non-small cell lung cancer (NSCLC). NSCLC, constituting over 80% of all lung cancer cases, arises from the uncontrolled proliferation of abnormal cells. Small-cell lung cancer has the potential to metastasize to other parts of the body. SCLC can be in two subgroups: small-cell carcinoma and combined small-cell carcinoma. While small-cell carcinoma is prevalent, combined small-cell carcinoma encompasses both NSCLC and SCLC. Notably, NSCLC is generally considered more hazardous than SCLC. NSCLC stages are adenocarcinoma, large-cell carcinoma, and squamous-cell carcinoma. The critical differentiator among these NSCLCs and SCLCs lies in their respective levels of aggressiveness. Various categories exist for respiratory system diseases, including emphysema and chronic bronchitis, both constituents of Chronic Obstructive Pulmonary Disease (COPD) [3]. Frequently co-occurring, these conditions contribute to the complex syndrome of COPD. Smoking is a predominant factor leading to obstructive pulmonary disease.

Chronic bronchitis induces inflammation and damage to the bronchial membranes connecting the lungs to the airways, resulting in persistent cough, heightened mucus production, and reduced air volume [4]. Emphysema, frequently coexisting with chronic bronchitis, is characterized by sneezing, shortness of breath, and diminished functional capacity. Asthma, a persistent disorder affecting the lungs and bronchi, primarily manifests through symptoms of wheezing and difficulty in oxygen inhalation. Cystic fibrosis, an inherited condition, disrupts mucus and sweat production, leading to recurrent lung infections and progressive, irreversible damage, culminating in severe respiratory failure. Tuberculosis, caused by a bacterium, predominantly affects the lungs, causing inflammation and subsequent destruction of lung tissue. Pneumonia encompasses a broad spectrum of infectious illnesses resulting from lung infections caused by diverse organisms, including parasites, viruses, bacterial infections, and fungi. Lung cancer, a significant contributor to cancer-related mortality affecting both genders, surpasses the combined fatalities resulting from breast, colon, and cervical cancers. Persistent coughing, often accompanied by chronic obstructive pulmonary disease, serves as a prevalent indication of lung cancer. Additional manifestations include expectations, chest pain, shortness of breath, appetite loss, weight loss, cold symptoms, and instances of bleeding [5].

The latest World Health Organization (WHO) records reveal that, in 2020, 12,174 deaths due to lung cancer [6]. As evidenced by WHO data, early cancer detection increases life expectancy. The prognosis for lung cancer is a complex task, contingent upon the tumor stage at diagnosis, characterized by uncontrolled cellular tissue growth.

The diagnostic process for lung cancer commonly involves chest CT scans and X-rays, with PET (Positron Emission Tomography) and MRI (Magnetic Resonance Imaging) occasionally employed to assess the extent of cancer metastasis. This comprehensive evaluation aids in formulating optimal therapeutic approaches. Bronchoscopy and biopsy, whether surgical or aspirational, are imperative for obtaining a precise diagnosis and identifying the histological type of lung cancer.

The prevalence of lung cancer is notably high among former smokers, underscoring the diverse risks associated with this population. Every smoker faces the potential risk of developing lung cancer over their lifetime. However, the prognosis in the later stages is grim, with a median survival rate of fewer than two years. Timely detection significantly improves the prognosis, with early-stage lung cancer presenting a favorable likelihood of cure [7]. Conversely, lung cancer identified at advanced stages typically results in a median survival period of fewer than two years.

Deep learning is playing transformative roles in addressing a critical concern— the prediction of lung cancer. Integrating data mining and deep learning approaches has become imperative in healthcare. Establishing comprehensive criteria is essential to guide and encourage the organic evolution of software tools grounded in artificial intelligence for the early prediction and detection of diseases. Applying artificial intelligence, machine learning, and deep learning proves beneficial in estimating the risk of various health conditions. This inclusive utilization of advanced techniques provides accurate methodologies for predicting lung cancer at its early stages. A chest X-ray or a low-dose CT scan is performed during the first assessment to discover lung anomalies. If there are any questionable indications, a PET scan is to assess the metabolic activity of the tumor and help distinguish between benign and malignant tumors [8].

In this study, we propose the implementation of knowledge distillation, augmented with additional teaching assistant models, for tumor detection in NSCLC. Traditional convolutional neural network (CNN) models, including CNN, ResNet, and VGG, alongside contemporary architectural designs such as Vision Transformer (ViT), Swin, and CCT, will undergo training for the knowledge distillation selective models. Hyperparameter tuning, adjusting parameters like alpha and temperature, will be undertaken to optimize the performance of the distilled student model. Furthermore, we have applied explainable AI techniques, leveraging a partition explainer, to examine the Shapley values and enhance the interpretability of the model’s predictions. Finally, a web application is developed for the lung images, particularly those likely unseen, allowing for practical implementation and validation of the proposed approach. The significant contribution of this work is as follows:

- Cost-sensitive learning approach is used to address the class imbalance issue of the employed NSCLC-Radiomics dataset.
- An intermediate model with ResNet152v2, known as teaching assistant, is added for the smooth transfer of knowledge from teacher (ViT) to student (custom CNN) with an intermediate step for the applied Knowledge Distillation technique. The additional teaching assistant model improves the performance of the Student model with distillation steps.
- The explainable AI approach, utilizing a partition explainer alongside SHAP, JSON, and other libraries, enhances the interpretability of predictions made by the deployed deep learning models.
- A user-friendly web application is developed to provide a practical interface for evaluating the finalized model’s performance on real-world lung cancer images.

The novelty of this work lies in integrating teacher, TA, and student-based three-phase explainable knowledge distillation techniques, which significantly reduce the training time for predicting non-small cell lung cancer.

## Related work

In recent years, plenty of studies have been performed to advance the predictive capabilities for automatic medical diagnosis of different complex diseases [9], especially lung cancer. The analysis of medical image datasets necessitates the expertise of qualified researchers [10]. The intricacies surrounding the symptoms, diagnosis, and treatment of lung cancer underscore the substantial costs, time investments, and susceptibility to resource constraints and human error. Notably, the focus of these studies ranges from tumor-specific analyses to the classification and segmentation of lung cancer types, encompassing both small and non-small cell categorizations. A brief overview of some studies within this context is presented in the following paragraphs. Hong et al. [11] applied CNN models to classify the type of lung disease in the dataset. Tuberculosis, pneumonia, pneumothorax types of lung disease, and other Normal lungs were classified. Images were to be trimmed at a ratio of 1:1 by 87.5% during preprocessing. EfficientNetB7 was selected to fit and evaluate the image dataset. 85.32% accuracy was best measured with the NIH image dataset. Pradhan and his co-authors [12] used 3D CNNs and CT scans to identify and detect lung cancer. The authors utilized the SPIE-AAPM dataset and removed lung nodules using preprocessing techniques. They achieved high levels of accuracy, scoring 100% on testing and 83.33% on training. Humayun et al. [13] built a system to categorize types of lung cancer. Applying a TL technique, they created a categorization system for patients’ lung cancer stages. As TL techniques, they employed VGG16, VGG19, and Xception. VGG16 and Xception achieved 98.83% and 97.4% accuracies, respectively. Kriegsmann and his co-authors [14] utilized CNNs to categorize common lung cancer subtypes, including SCLC, ADC, and SqCC, and established quality control methods to identify patients for further investigation. With the NCT’s Tissue Biobank, the University Clinic Heidelberg’s Institute of Pathology cataloged 80 lung cancer types. Primakov et al. [15] implemented a fully automated system for detecting and volumetrically segmenting non-small cell carcinoma of the lung on CT images of the thorax. The authors created a three-step methodology, including image pre-processing, lung separation, and automated tumor identification and segmentation. Their recommended technique offers excellent tumor-detection sensitivity (0.97) and specificity (0.99), according to the area beneath the curve of 0.98. Tandon and team [16] introduced VCNet, a hybrid model for detecting cancerous lung nodules in CT scans. The VCNet model combines the capabilities of the VGG-16 and the capsule network (CapsNet). VGG-16 is used for object detection and classification, while CapsNet assists with picture rotation, tiling, and anomalous orientations. For the LIDC dataset, the model accomplished a testing accuracy of 99.49%, surpassing competing models such as MobileNet, Xception, and VGG-16. Tyagi et al. [17] combined CNN techniques with vision transformers for autonomous lung tumor segregation. This model was trained on the NSCLC-Radiomics dataset, and its generalizability was verified using data from a local hospital. They found average dice coefficients of 0.7468 and 0.6847, respectively, and Hausdorff distances of 15.336 and 17.435. Chen and his colleagues [18] used the Swin Transformer to classify lung cancer. Under bronchoscopic supervision, patients underwent interventional cytology, utilizing 347 photographs of lung washout cells, resulting in 2473 images of individual cell nuclei, enhancing the study’s findings. The experimental results revealed an impressive classification accuracy rate of 96.16%. Zheng et al. [19] developed an effective method for classifying lung tumor surgical specimen sections’ images using knowledge distillation. A clinical lung tumor public datasets, i.e., LIDC-IDRI and LUNA16, were used. The applied ConvNeXt-based KD model produced a better classification accuracy of 85.64% and an F1 score of 0.7717. Sun and his colleagues [20] developed an effective method for segmenting and classifying lung cancer images based on an enhanced Swin transformer and LUNA16 dataset. The pre-trained Swin-B model outperformed ViT by 2.529% with an accuracy of 82.26% in the classification task. Cao and colleagues [21] created an efficient way to identify lung nodules through the 3D multidimensional attention encoder-decoder networks. Cao and colleagues [22] used a multi-scale MobileViT for pulmonary nodule classification with a dataset from LIDC-IDRI that included 442 benign and 406 malignant nodules. The researchers used a CNN structure with sub-pixel fusion, dilated convolution, and the MobileViT module. This strategic application of MobileViT improved classification results, with the best accuracy of 94.04% and an AUC value of 0.9636 after ten-fold cross-validations with the constraints of the dataset and MobileViT’s specific advantages.

Uzelaltinbulat et al. [23] introduced a novel algorithmic method to medical image processing for the segmentation of lung cancers in CT images. The methodology comprised several stages of automatic threshold selection, image subtraction for unique tumor segmentation, and image pre-processing with noise reduction algorithms. An extensive evaluation of the suggested approach was conducted with a dataset from the NIH/NCI Lung Image Database Consortium, demonstrating a high accuracy of 97.14%, with 100% and 96% sensitivity and specificity, respectively. Kim and his teammates [24] proposed a transfer learning framework, Response-based Cross-task Knowledge Distillation (RCKD), for pathological image analysis. RCKD pretrains a student model for predicting nuclei segmentation in pathological images, fine-tuning it for tasks like organ cancer sub-type classification and cancer region segmentation. The RCKD model achieved 94.2% accuracy on six pathological image datasets, 4% and 7.4% higher than EfficientNet-B0 and ConvNextV2, respectively.

Fangxing et al. [25] applied a ResNet-18-based pretrained knowledge distillation model for lung cancer identification. When applied to an open-source dataset of lung tissue categories, their lightweight deep learning approach achieved significant size reduction while maintaining exceptional performance metrics. The distilled model reduced 55.15% of its parameters and attained excellent classification accuracy. Chen and colleagues [26] developed a method to correlate CT images with pathological examination results for lung adenocarcinoma diagnosis. The authors utilized four datasets, with datasets 1 and 2 from local hospitals and datasets 3 and 4 from online repositories. The computational study validated the method’s reliability in aiding adenocarcinoma diagnosis, with dataset 1 demonstrating the highest performance, reaching 97.9% accuracy and a 96.9% AUC.

Dong et al. [27] introduced a novel multi-view information integration and propagation mechanism to mitigate model disturbances caused by occlusion noise in re-identification tasks. Additionally, the authors devised localization and quantification modules incorporating distillation techniques to counteract occlusion noise effects. Their study involved a comparative evaluation with state-of-the-art methods using five publicly available person re-identification datasets, including O-Duke and P-Duke. Yan and his coauthors [28] applied the multilevel alignment network (MANet) for text-based person searches. They implemented the local and global alignment modules to enhance semantic alignment between aggregation features. The proposed method was evaluated on the CUHK-PEDES dataset, which comprises 54,522 images of 4,102 individuals. MANet achieved an inference time of 15.422s, comparable to the Baseline and GA methods. Li et al. [29] employed the knowledge-guided semantic transfer network (KSTNet) for few-shot image recognition. The KSTNet leverages knowledge transfer and learning from classifiers to develop a robust semantic visual mapping. Their approach was evaluated on two publicly available ImageNet datasets, yielding promising results.

Tang et al. [30] introduced an attention-guided bidirectional pyramid architecture to enrich feature representation while effectively mitigating background-induced uncertainty. The study encompassed the exploration of four widely recognized fine-grained datasets. Their approach notably demonstrated a significant enhancement, achieving a 7.12% and 5.77% improvement solely through the utilization of the pyramidal architecture. Tang, with his coauthors [31], applied the meta-regularization method along with Blockmix and proposed a novel inference scheme called self-calibrated inference for metric-based meta-learning. The experiments were conducted using the MiniImageNet and CUB-200-2011 datasets under two settings, i.e., 1-shot and 5-shot classification. The results demonstrated superior performance in 1-shot classification compared to 5-shot classification tasks.

He et al. [32] utilized DNA somatic mutation data from 4,909 samples spanning 13 cancer types to determine the tissue-of-origin for carcinoma of unknown primary. The random forest approach produced an F1 score of 0.8886 and an average accuracy of 0.8822 using a 600-gene set. This approach works better than conventional imaging modalities. Chen and colleagues [33] investigated the potential synergistic effects of taxol and purvalanol A, two Cdc2/Cdk1 inhibitors, on boosting apoptosis in NSCLC cells. The authors showed that purvalanol A decreases cellular proliferation and colony formation and increases taxol-induced apoptosis using NCI-H1299 and CNE1 cell lines. In addition to drastically reducing Bcl-2 expression and phosphorylating Op18/stathmin, a protein linked to taxol resistance, the combination therapy also activates caspase-3 and caspase-8.

Researchers have utilized various deep learning frameworks and diverse preprocessing methodologies to classify distinct types of lung cancer accurately. However, the related works involve inherent limitations, such as insufficient quantities and uneven distribution of images, excessive trainable parameters leading to high training time and memory requirements, etc. Motivated by these considerations, this work implements knowledge distillation techniques, enriched by incorporating data balancing techniques with cost-sensitive learning and an additional teaching assistant model with ViT-ResNet152v2-CNN framework, contributing significantly to model size reduction and training time. Furthermore, an explainable AI technique is applied to predict the Shapley values for multiclass to assess the importance of the contribution of each class.

## Materials and methods

**Fig 1:**
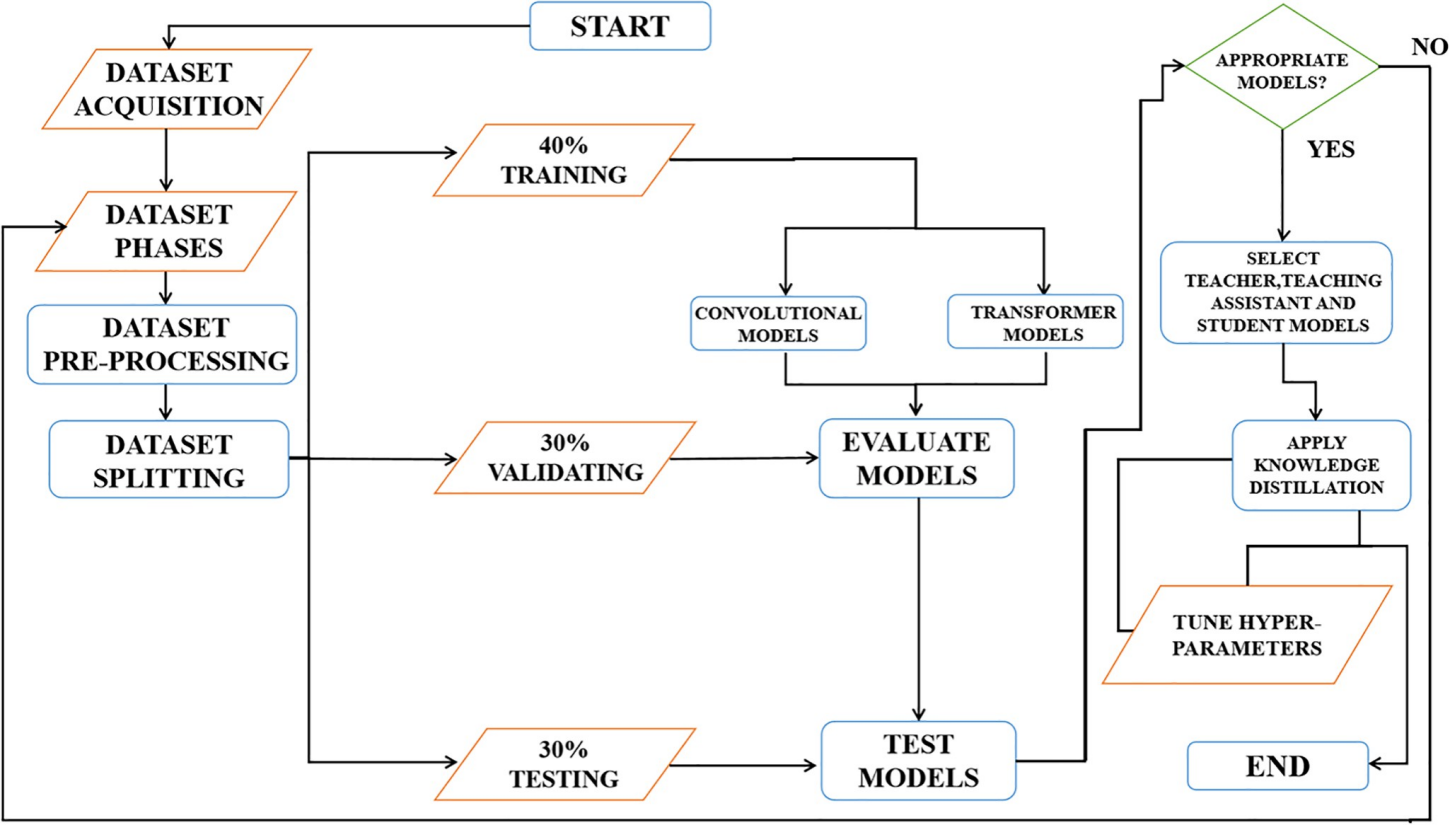
Working sequences of the proposed NSCLC classification system.

Fig 1 depicts the working sequences of the proposed NSCLC detection system. The implementation details of the applied steps are discussed in the subsequent paragraphs.

### Dataset

In this work, we utilized an enhanced version of a publicly available dataset known as NSCLC-Radiomics, initially introduced in [34]. This dataset consists of 51,215 CT scan images from 422 NSCLC patients. These images are categorized into five classes. Among the classes, three are non-small cell lung cancer types. The other two classes are mixed-type and normal/healthy lungs. Table 1 shows the total number of images and percentage occupied according to classes.

**Table 1:** Details of the NSCLC-Radiomics dataset.

Class	Number of Images	Percentage Occupied
Adenocarcinoma	6018	11.75%
Large cell carcinoma	13655	26.66%
Normal	5130	10.01%
Not otherwise specified	7643	14.92%
Squamous cell carcinoma	18769	36.64%

**Fig 2:**
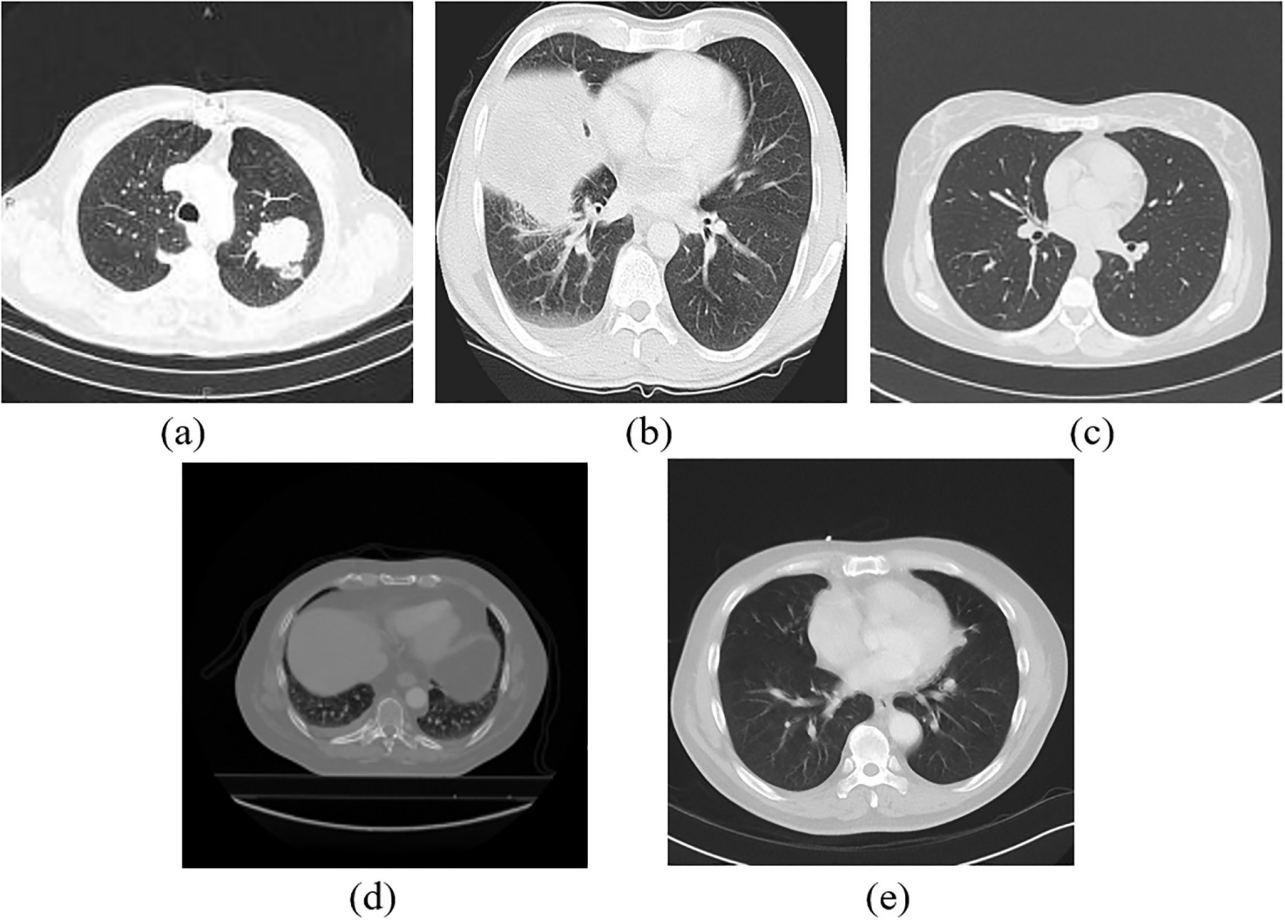
Sample images of various classes: (a) Adenocarcinoma, (b) Large cell carcinoma, (c) Normal, (d) Not otherwise specified, (e) Squamous cell carcinoma.

The dataset is not balanced, according to Table 1. Squamous cell carcinoma accounts for more than 30% of all images, while large cell carcinoma accounts for less than 20%. Fig 2 shows sample images from five different classes of the NSCLC-Radiomics dataset.

#### Dataset preprocessing

In this research, various preprocessing steps have been performed to make the images ready to fit and evaluate in the model. The images are resized to 80 × 80 pixels. Each model recommends a different size of pixels. But we have set every model to a fixed size to make them comparable. We have also trimmed the 10% side of all images to make them fit the model. Next, cropping the central area of images by 90% is done. The photos have been renamed in the following format: *class*_*id*. In this case, the class represents one of the five classes, and *id* is a unique number assigned to each image. BulkRenameUtility tool has been employed to rename files in this work. Utilizing BulkRenameUtility, we changed all photographs to .png format since it allows for translucent backgrounds and retains the original image quality. We used normalization to close pixel values ranging from 0 to 1.

#### Cost-sensitive learning

Cost-sensitive learning is a preprocessing technique to handle dataset imbalance issues. Assigning higher costs to the minority class and vice versa improves the overall model performance of an imbalanced dataset. It assigns distinct misclassification costs to different classes as:

$$\begin{array}{c} Class\;Weight=\frac{Total\;Samples}{Total\;Classes\times Total\;Samples} \end{array}$$ (1)

### Applied models

#### Modern transformers

- **Swin:** Swin transformer refers to a shifted window transformer that brings greater efficiency by limiting self-attention computation to non-overlapping local windows [35]. This architecture has the flexibility to model information at various scales. This architectural design provides benefits to all MLP architectures.
- **ViT:** Vision transformer (ViT) was developed for natural language processing tasks [36]. It provides such functionality that images are converted into patches. These patches are linearly embedded in high-dimensional vectors, formatting the input into a transformer model. It has demonstrated competitive performance with CNN on various computer vision benchmarks.
- **CCT:** The compact convolution transformer (CCT) is another modern design that makes use of convolution. Compact convolutional transformers use an all-convolution mini-network to generate picture patches [37]. It not only uses sequence pooling, but it also replaces the patch embedding with a convolutional embedding, allowing for improved inductive bias and making positional embeddings unnecessary.

#### Knowledge distillation with teaching assistant

Knowledge distillation is the process of distilling knowledge from a large model to a teacher to a smaller one, like a student [38]. While huge models (such as very deep neural networks or ensembles of numerous models) have greater knowledge capacity than small models, this potential may not be completely utilized. The large model is relatively complex, so distilling knowledge results in a compact and efficient student model [39]. The teacher’s soft predictions (probabilities) can be used as “soft targets” during training, allowing the student model to learn from more robust and less noisy labels, especially when the training labels contain errors. In this study, we have added an extra model denoted as teaching assistant [40], which is in the intermediate model between the teacher and student model. Usually, in a knowledge distillation technique, the teacher trains a subset of the dataset to gain knowledge. Next, the student model is distilled and then fits and evaluates this model. The teaching assistant model adds an extra advantage to the student model. At first, the teacher distills the teaching model, and then the teaching assistant model works the same way as the teacher model. This model trains a subset of the dataset and distills it to the student model. Student models gain knowledge from the teaching assistant rather than the teacher model. It builds a robust, compact, reliant, and efficient student model that performs better than before approach.

**Fig 3:**
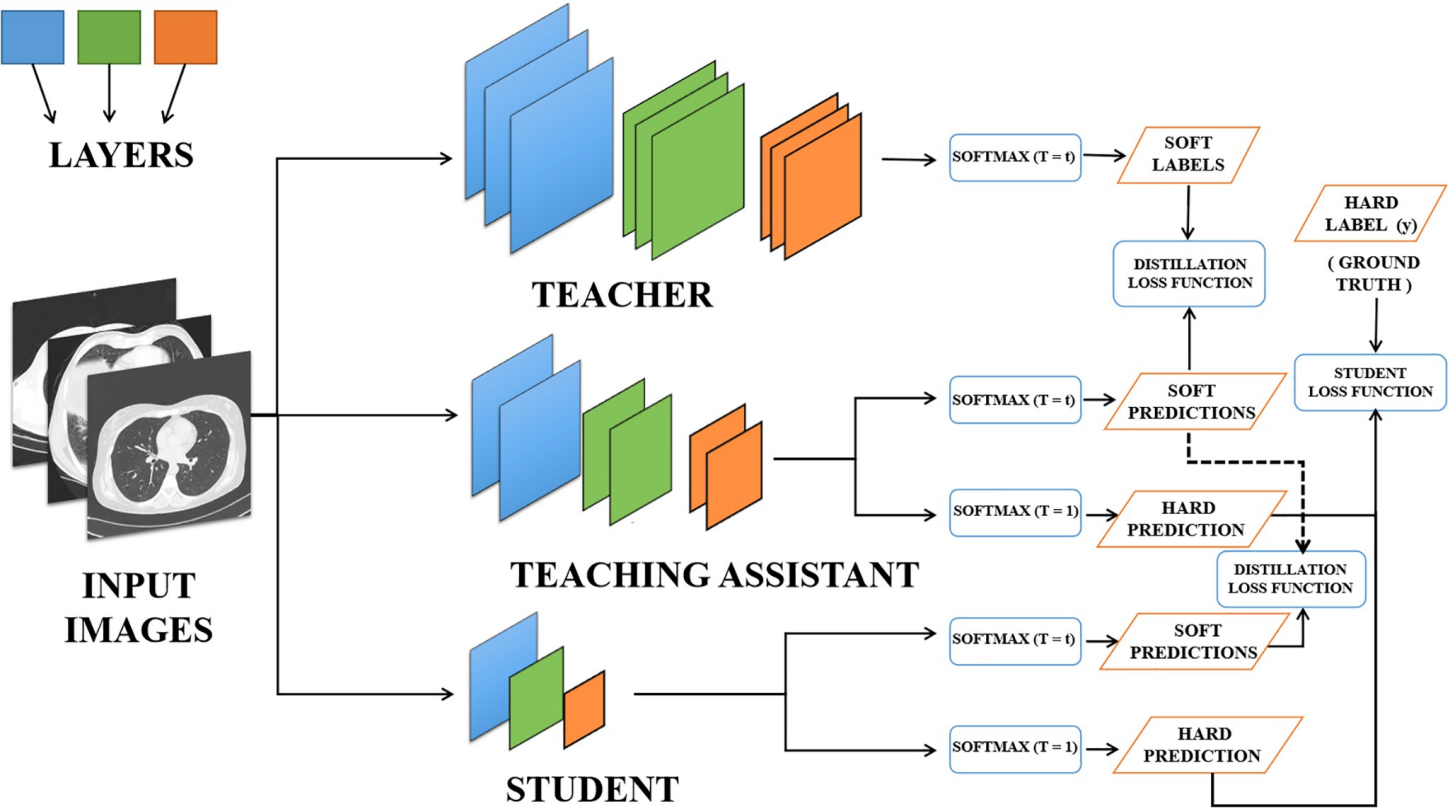
Knowledge distillation architecture used in this work.

Fig 3 illustrates the knowledge distillation architecture proposed in this work. Input images are fed into all models. Initially, the teacher model generates predictions employing softmax layers to produce soft labels. Following the training of the Teacher model, it imparts its knowledge to the teaching assistant model. The teaching assistant model subsequently makes soft predictions utilizing the distilled knowledge from the Teacher model while also being trained on the ground truth or hard labels. Upon completion of training in the teaching assistant model, the Student model is distilled from it. The Student model, in turn, generates soft predictions based on the predicted teaching assistant labels. Additionally, Student models have the option to train on hard labels or ground truth for producing hard predictions. This sequential process of knowledge distillation ensures that the learned knowledge progressively transfers from the teacher model to the teaching assistant model and finally to the student model. The use of soft labels at each stage facilitates a more nuanced understanding of the data, contributing to the overall enhancement of the Student model’s performance.

After evaluating all models in each phase, we selected three models suitable for applying knowledge distillation according to complexity. Table 2 represents the overall architecture of the Vision transformer model, which we selected as the Teacher model. Table 3 represents the teaching assistant model, for which we selected the ResNet152v2 traditional convolutional model. Lastly, we selected CNN as the Student model, which we assumed was simple and compact, represented in Table 4.

**Table 2:** ViT (Teacher) model’s summary.

Layer Name	Input Shape	Output Shape
input_15	(None, 80, 80, 3)	(None, 80, 80, 3)
patches_8	(None, 80, 80, 3)	(None, None, 432)
patch_encoder_8	(None, None, 432)	(None, 36, 1024)
layer_normalization_488	(None, 36, 1024)	(None, 36, 1024)
multi_head_attention_240	(None, 36, 1024)	(None, 36, 1024)
add_480	(None, 36, 1024)	
layer_normalization_489	(None, 36, 1024)	(None, 36, 1024)
dropout_268	(None, 36, 1024)	(None, 36, 1024)
add_481	(None, 36, 1024)	
layer_normalization_490	(None, 36, 1024)	(None, 36, 1024)
multi_head_attention_241	(None, 36, 1024)	(None, 36, 1024)
add_482	(None, 36, 1024)	
layer_normalization_491	(None, 36, 1024)	(None, 36, 1024)
dropout_269	(None, 36, 1024)	(None, 36, 1024)
add_483	(None, 36, 1024)	
layer_normalization_492	(None, 36, 1024)	(None, 36, 1024)
multi_head_attention_242	(None, 36, 1024)	(None, 36, 1024)
add_484	(None, 36, 1024)	
layer_normalization_493	(None, 36, 1024)	(None, 36, 1024)
dropout_270	(None, 36, 1024)	(None, 36, 1024)
add_485	(None, 36, 1024)	
layer_normalization_494	(None, 36, 1024)	(None, 36, 1024)
multi_head_attention_243	(None, 36, 1024)	(None, 36, 1024)
add_486	(None, 36, 1024)	
layer_normalization_495	(None, 36, 1024)	(None, 36, 1024)
dropout_271	(None, 36, 1024)	(None, 36, 1024)
add_487	(None, 36, 1024)	
layer_normalization_496	(None, 36, 1024)	(None, 36, 1024)
multi_head_attention_244	(None, 36, 1024)	(None, 36, 1024)
add_488	(None, 36, 1024)	
layer_normalization_497	(None, 36, 1024)	(None, 36, 1024)
dropout_272	(None, 36, 1024)	(None, 36, 1024)
add_489	(None, 36, 1024)	
layer_normalization_498	(None, 36, 1024)	(None, 36, 1024)
flatten_16	(None, 36, 1024)	(None, 36864)
dropout_273	(None, 36864)	(None, 36864)
dense_53	(None, 36864)	(None, 256)
dropout_274	(None, 256)	(None, 256)
dense_54	(None, 256)	(None, 128)
dense_55	(None, 128)	(None, 5)
**Total Trainable Parameters:**		378,269,216
**Total Non-trainable Parameters:**		4,096
**Total Parameters:**		378,273,312

**Table 3:** ResNet152v2 (teaching assistant) model’s summary.

Layer Name	Input Shape	Output Shape
resnet152v2_input	(None, 80, 80, 3)	(None, 80, 80, 3)
resnet152v2	(None, 80, 80, 3)	(None, 3, 3, 2048)
gaussian_noise_6	(None, 3, 3, 2048)	(None, 3, 3, 2048)
global_average	(None, 3, 3, 2048)	(None, 2048)
pooling2d_3		
dense_38	(None, 2048)	(None, 128)
batch	(None, 128)	(None, 128)
normalization_3		
gaussian_noise_7	(None, 128)	(None, 128)
dropout_246	(None, 128)	(None, 128)
flatten_12	(None, 128)	(None, 128)
dense_39	(None, 128)	(None, 5)
**Total Trainable Parameters:**		147,603,456
**Total Non-trainable Parameters:**		3,984
**Total Parameters:**		147,607,440

**Table 4:** CNN (Student) model’s summary.

Layer Name	Input Shape	Output Shape
conv2d_3_input	(None, 80, 80, 3)	(None, 80, 80, 3)
conv2d_3	(None, 80, 80, 3)	(None, 78, 78, 64)
max_pooling2d_15	(None, 78, 78, 64)	(None, 39, 39, 64)
flatten_10	(None, 39, 39, 64)	(None, 97344)
dense_30	(None, 97344)	(None, 256)
dropout_236	(None, 256)	(None, 256)
dense_31	(None, 256)	(None, 128)
dropout_237	(None, 128)	(None, 128)
dense_32	(None, 128)	(None, 64)
dropout_238	(None, 64)	(None, 64)
dense_33	(None, 64)	(None, 5)
**Total Trainable Parameters:**		25,009,500
**Total Non-trainable Parameters:**		3,792
**Total Parameters:**		25,013,292

#### Explainable artificial intelligence

Explainable artificial intelligence (XAI) allows users to analyze and trust the results, output, and overall performance created by the applied AI algorithms. It describes impacts and characterizes accuracy and fairness in AI decision-making. In image classification, XAI is used for feature visualization to show how features contribute to prediction and overall model performance. Using SHAP values, we can interpret the feature’s importance and attribute the model’s decision to different input image pixels.

## Results and discussion

In this study, we employ Anaconda Navigator, an open-source Python distribution designed for streamlined package management and deployment in data research. This comprehensive platform encompasses various tools, including Jupyter Notebook, Spyder, and JupyterLab. Notably, Jupyter Notebook, our primary Python Integrated Development Environment (IDE) for data science, serves as the tool for training and testing our dataset. In addition, we employ a Google-supplied cloud-based platform that provides a conducive environment for Python development. The tasks undertaken in this study were executed on a dedicated computing device—Processor: Intel(R) Core(TM) i5-3427U CPU @ 1.80GHz, RAM: 4.00 GB DDR3-2133 SDRAM, System Type: 64-bit operating system, x64-based processor. We have used the KL divergence loss function for distillation loss and the Categorical cross-entropy function for student loss. In this research, Adam optimizer, ReLU activation function, 100 training epochs, 0.001 learning rate and batch size 64 are employed to train the applied models.

After preprocessing, we partitioned the dataset into three subsets using a 4:3:3 ratio, with 40% of the data designated for the training set and the remaining 30% allocated to both the test and validation sets. Given the inherent imbalance in our dataset regarding class distribution, we carefully considered representative images across all classes. The dataset comprises 51,215 images, with approximately 20,000 in the training folder, while the validation and test folders each contain close to 15,000 images. After partitioning the dataset, we proceeded with fitting and evaluating the models. Despite varying recommendations for different models, we standardized the image resolution to 80 × 80 across all models. With a constant batch size of 64, a uniform resolution was applied for both convolutional and transformer models, and specialized approaches customized to each model were used to address overfitting and minimize vector dimensions. Subsequently, we incorporated knowledge distillation methodologies, integrated partition explainer-based XAI framework, and developed a web application for predicting outcomes on previously unseen images.

### Baseline (undistilled) models

Initially, a wide range of baseline (without employing knowledge distillation techniques) models and cost-sensitive learning approaches are employed for the proposed lung cancer classification. The corresponding weights for each category of cancer are calculated using (1) as:

$$\begin{array}{c} Adenocarcinoma=\frac{51215}{5\times 6018}=1.70 \end{array}$$ (2)

$$\begin{array}{c} Large\;Cell\;Carcinoma=\frac{51215}{5\times 13655}=0.75 \end{array}$$ (3)

$$\begin{array}{c} Normal=\frac{51215}{5\times 5130}=1.99 \end{array}$$ (4)

$$\begin{array}{c} Not\;Otherwise\;Specified=\frac{51215}{5\times 7643}=1.34 \end{array}$$ (5)

$$\begin{array}{c} Squamous\;Cell\;Carcinoma=\frac{51215}{5\times 18769}=0.54 \end{array}$$ (6)

Table 5 presents various performance metrics of the applied baseline (undistilled) models. According to this table, the ViT-based transformer model accomplished the best accuracy of 95.67%. The proposed custombuilt CNN and ResNet152v2 techniques achieved accuracies of 95.27% and 95.16%, respectively. Notably, all the applied models are trained with 200 epochs and executed on the Google Colaboratory Professional Plus platform, employing the A100 GPU.

**Table 5:** Performance metrics of the applied baseline (undistilled) models.

Models	Accuracy	Precision	Recall	Training time per epoch
**CNN**	95.27%	95.62%	94.93%	25.15s
**ResNet152v2**	95.16%	95.45%	95.07%	191.11s
**VGG19**	94.31%	94.47%	94.23%	100.15s
**Swin**	61.35%	78.67%	43.72%	560.5s
**ViT**	95.67%	95.99%	95.35%	65.3s
**CCT**	64.55%	77.16%	49.23%	30.05s

### Knowledge distillation models

**Fig 4:**
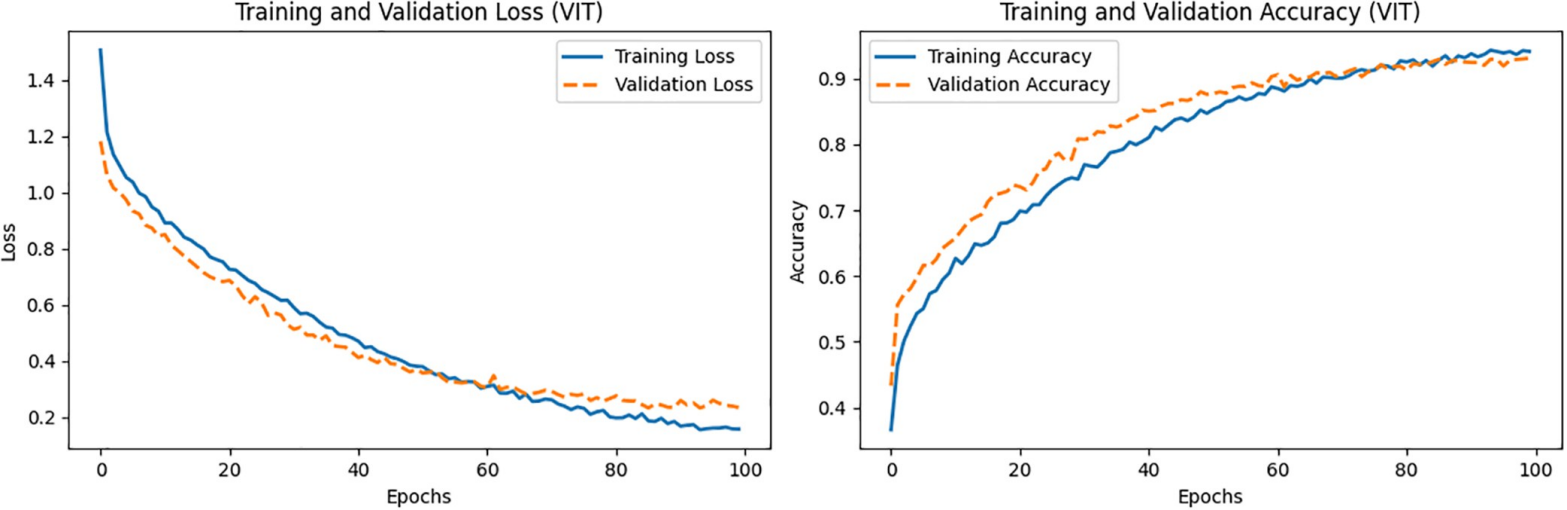
Loss and accuracy vs. epochs of the ViT (teacher) model.

In this work, ViT was chosen as the teacher model because of its superior performance compared to other models. ResNet152v2 was selected as the teaching assistant (TA) model due to its versatility across different phases, demonstrating excellent performance. Because of its simplicity and notable performance on the employed dataset, a custom CNN was ultimately designated as the student model. Fig 4 illustrates the training and validation losses and accuracies with the change of epochs for the ViT-based teacher model. Various performance metrics of this model are listed in Table 6.

**Table 6:** ViT (Teacher) model’s performance metrics.

Metrics	Train	Validation	Test
Loss	0.1586	0.2352	0.2253
Categorical accuracy	94.14%	93.13%	93.31%
Precision	94.95%	94.45%	94.98%
Recall	93.19%	92.07%	91.80%
Cosine similarity	95.12%	93.96%	94.07%

We systematically tuned the hyperparameters of the proposed knowledge distillation approach, specifically alpha and temperature, to explore the varied outcomes of the distilled models. Alpha assigns weights to the distillation loss, while temperature primarily influences the softmax activation function, facilitating softer predictions. We selected alpha values of 0.3, 0.5, and 0.7 and temperature values of 7, 15, 22, and 30 to observe distinct results. The obtained results were juxtaposed with those of the baseline models associated with the designated hyperparameters. The distilled ResNet152v2-based TA model, as presented in Table 7, demonstrated an accuracy ranging between 80% and 91%, consistently lower than its baseline model. The student CNN model, outlined in Table 8, consistently achieved an accuracy exceeding 90% with a maximum of 94.53%, closely resembling its baseline model. Remarkably, the optimal combination of hyperparameters was identified as an alpha of 0.7 and a temperature of 7. This configuration yielded superior performance, with accuracy metrics reaching 90.99% and 94.53% for the distilled ResNet (TA) and distilled CNN (student) models, respectively.

**Table 7:** Distilled ResNet (TA) model’s performance with hyperparameter tuning.

Alpha	Temperature	Distilled ResNet Accuracy (%)	ResNet Accuracy (%)
0.3	7	85.71%	95.16%
	15	85.51%	
	22	86.19%	
	30	81.60%	
0.5	7	89.88%	
	15	88.35%	
	22	89.33%	
	30	87.55%	
0.7	7	90.99%	
	15	89.89%	
	22	81.41%	
	30	88.03%	

**Table 8:** Distilled CNN (student) model’s performance with hyperparameter tuning.

Alpha	Temperature	Distilled CNN Accuracy (%)	CNN Accuracy (%)
0.3	7	92.87%	95.27%
	15	91.60%	
	22	89.91%	
	30	93.39%	
0.3	7	93.83%	
	15	94.23%	
	22	93.77%	
	30	93.76%	
0.3	7	94.53%	
	15	88.00%	
	22	85.20%	
	30	93.97%	

**Fig 5:**
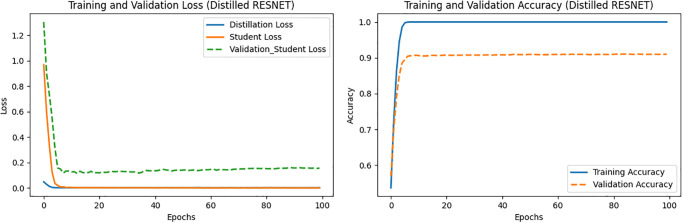
Loss and accuracy vs. epochs of the ResNet (TA) model.

**Fig 6:**
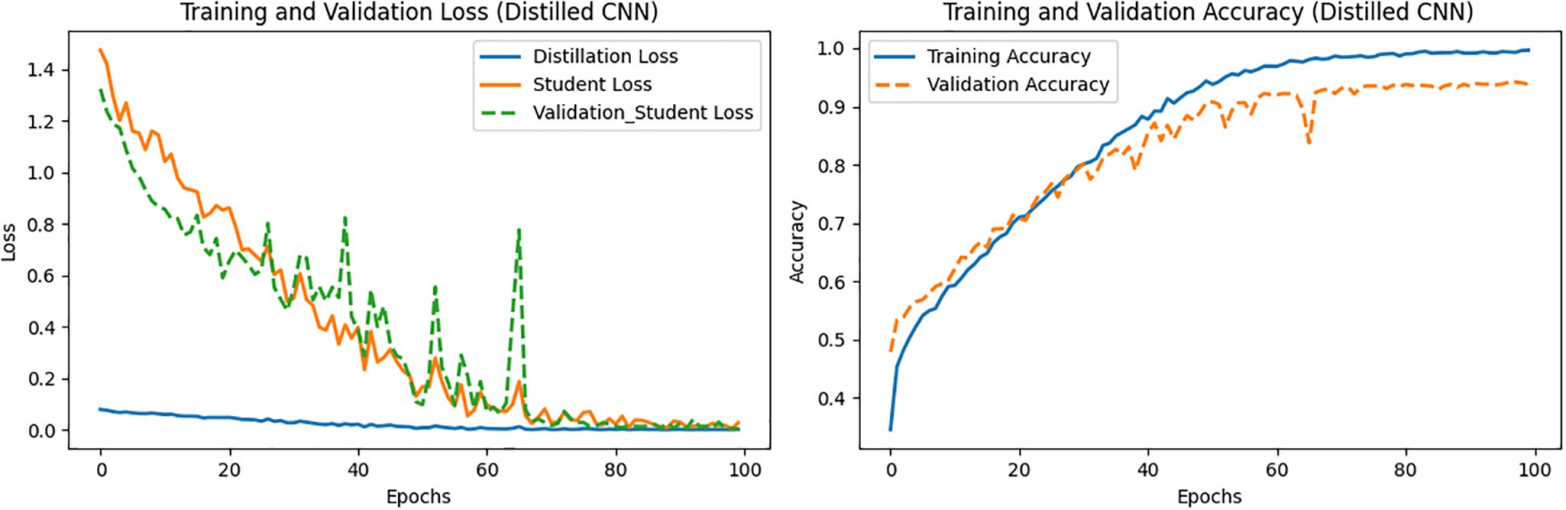
Loss and accuracy vs. epochs of the CNN (student) model.

With alpha and temperature values set to 0.7 and 7, respectively, the obtained training and validation losses and accuracies of the ResNet152-based TA and custom CNN-based student networks are depicted in Figs 5 and 6, respectively. Various performance metrics of the proposed TA and student models are listed in Tables 9 and 10, respectively.

**Table 9:** TA model’s performance metrics.

Metrics	Train	Validation	Test
Student Loss	3.7788e-04	0.1555	0.3028
Categorical acc.	100%	91.00%	90.99%
Precision	100%	91.74%	92.20%
Recall	100%	90.53%	90.09%
Cosine similarity	100%	92.10%	92.18%
Distillation Loss	(0.0011)		

**Table 10:** Student model’s performance metrics.

Metrics	Train	Validation	Test
Student Loss	0.0190	0.0020	0.2293
Categorical acc.	99.64%	93.76%	94.53%
Precision	99.72%	94.05%	94.92%
Recall	99.50%	93.55%	94.37%
Cosine similarity	99.62%	94.50%	95.08%
Distillation Loss	(8.3356e-04)		

Tables 11 and 12 present the statistics detailing the time elapsed for the applied ResNet152 (TA) and CNN (student) models, respectively. The reported metrics encompass the total training time, training time per epoch, and the specific GPU type utilized in this model. During the knowledge distillation process, a significant reduction in training time has been observed, resulting in performance increases of roughly 4% for the student model and 2% for the teaching assistant model. Additionally, this methodology reduces the number of parameters within the distilled model. Consequently, distilled models can attain a comparable level of performance with a decreased parameter count, offering potential advantages in resource-constrained environments.

**Table 11:** TA model’s execution time details.

Subset	GPU	Epochs	Time elapsed	Time per epoch
**Train**	A100 GPU	100	3756s	5.09s
**Validation**	with	100	3756s	5.09s
**Test**	high memory	-	10s	39ms

**Table 12:** Student model’s execution time details.

Subset	GPU	Epochs	Time elapsed	Time per epoch
**Train**	A100 GPU	100	501s	5.01s
**Validation**	with	100	501s	5.01s
**Test**	high memory	-	1s	6ms

**Fig 7:**
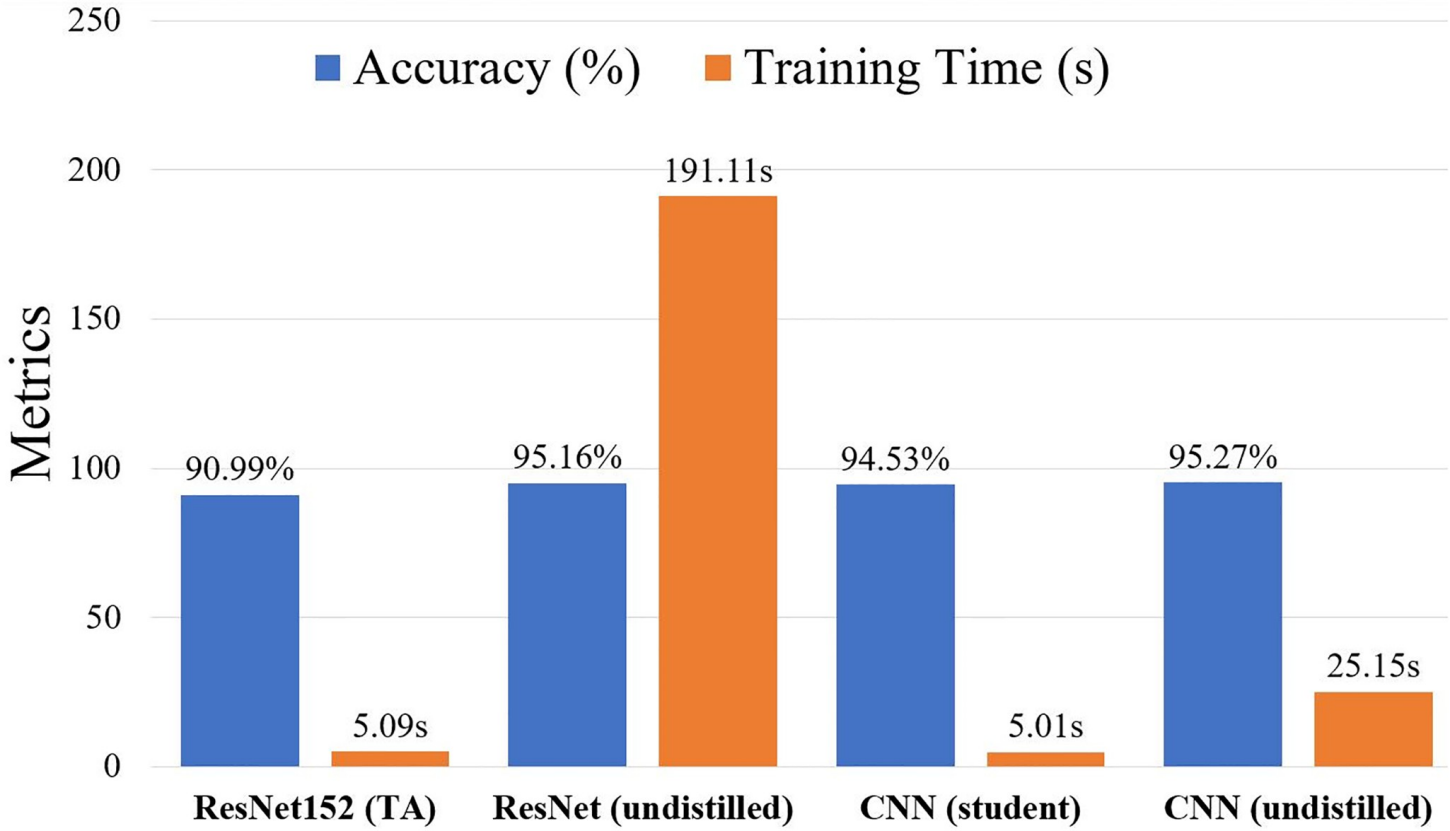
Accuracy and training time per epoch of the applied models.

Fig 7 summarizes the accuracy and training time per epoch for the applied models. It shows a 4.17% decrease in accuracy for the distilled ResNet152-based TA model. Conversely, the training time is reduced by a factor of 37.55 for this model, significantly reducing trainable parameters and memory usage. The CNN-based student model achieves a 5.02-fold reduction in training time compared to its undistilled baseline counterpart. Therefore, it can be concluded that the applied knowledge distillation techniques significantly reduce training time while maintaining excellent classification performance.

### Explainable AI using partition explainer

**Fig 8:**
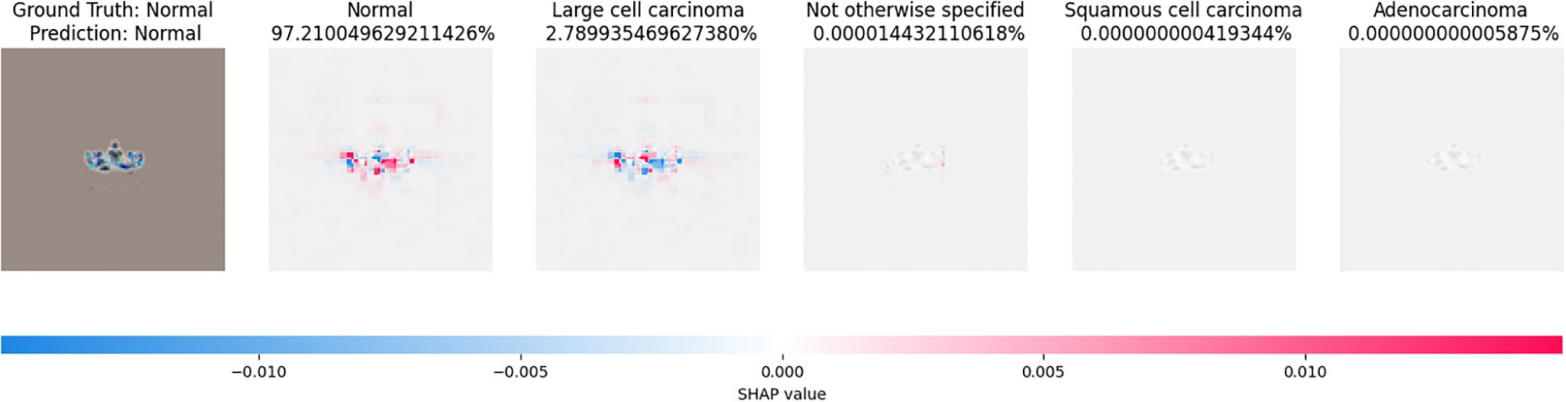
XAI interpretation of a test image.

After obtaining results from the knowledge distillation approach, we applied explainable artificial intelligence (XAI) to an unseen image for predicting Shapley values. We employed a partition explainer, incorporating the model, masker Python function, and class names. The masker, implemented as a Python function, utilizes a blurring technique. Subsequently, we utilized SHAP values to identify the most probable classes among all classes, as depicted in Fig 8.

Fig 8 illustrates the impact of the five classes on unseen images. Specifically, large-cell carcinoma exhibits higher positive values than squamous-cell carcinoma, while other categories demonstrate no discernible influence on the corresponding image.

### Web application implementation

**Fig 9:**
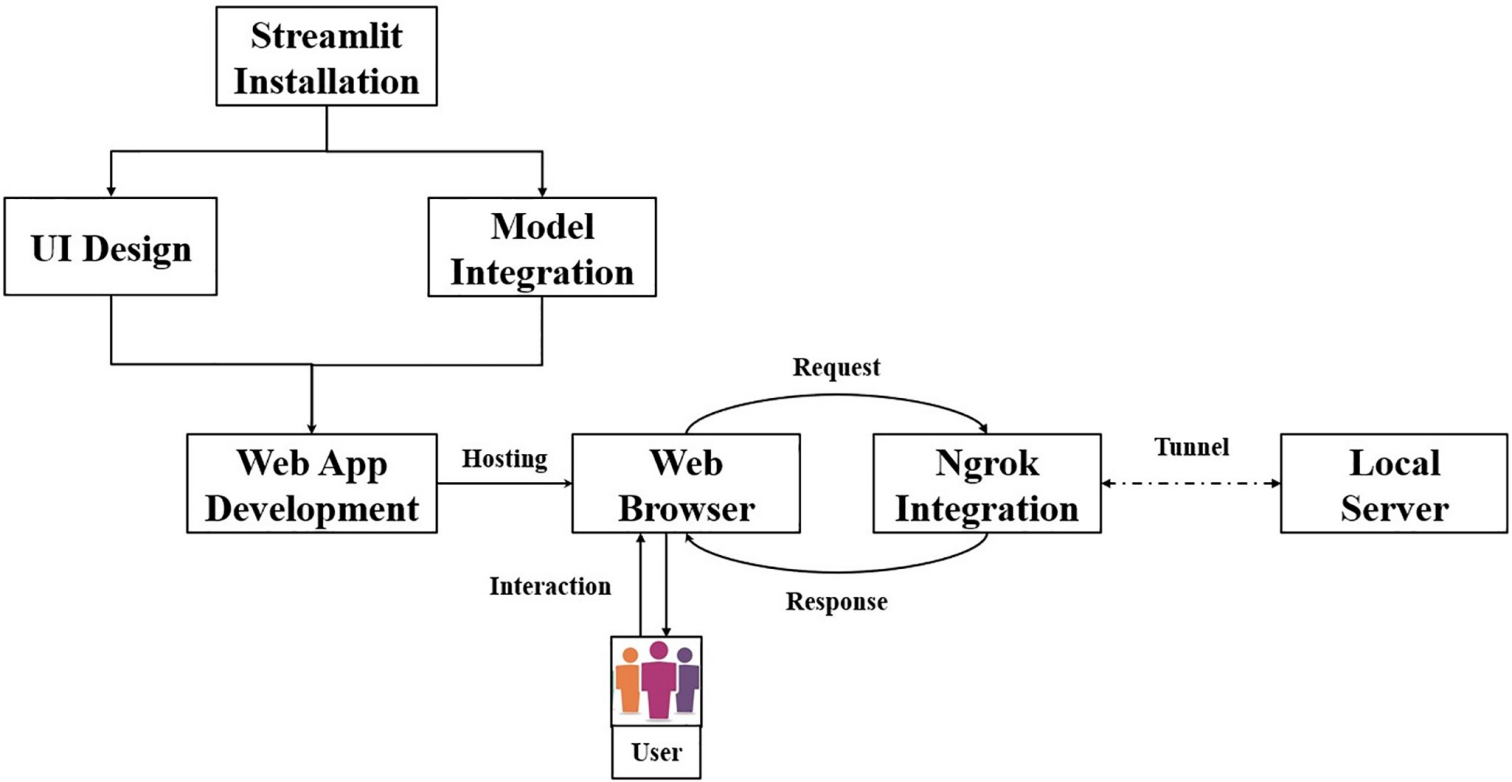
Development and deployment process of the proposed web application.

**Fig 10:**
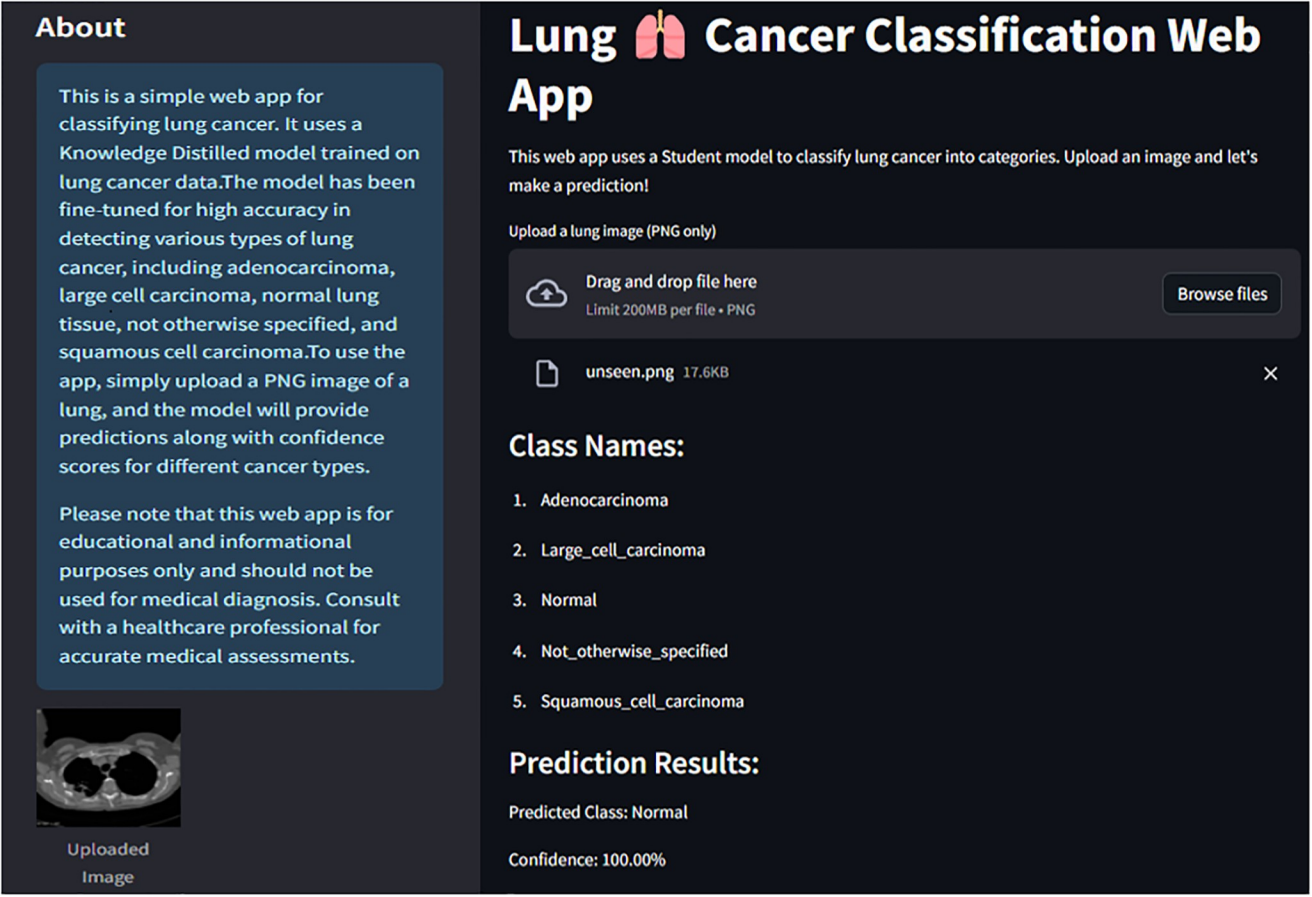
Web app implementation of the proposed system.

Finally, a web application has been developed to instantaneously predict the class of previously unseen images. The application incorporates the proposed student model based on distilled CNN architecture. Fig 9 illustrates developing and deploying the proposed web application using Streamlit, a widely used and intuitive framework for web application development. Initially, integrating Streamlit entails downloading and installing it within Google Collaboratory. Subsequently, leveraging Streamlit’s layout commands, e.g., “st.sidebar” and “st.columns,” facilitates the structuring of the application. The framework’s versatile components and widgets, encompassing sidebars, buttons, and text inputs, enable the creation of interactive elements to manage user inputs effectively. Notably, the layout has been meticulously crafted utilizing Streamlit’s theming options alongside CSS and HTML. Integration of the model with Google Collaboratory furnishes the capability to develop the web application adept at processing input images, executing preprocessing tasks, and delivering predictions or results seamlessly. The integration of Ngrok with Streamlit facilitates local hosting by establishing a secure tunnel to the local server, thereby rendering the application accessible over the internet via a unique URL. This streamlined interaction between users and the web application is achieved through standard web technologies such as HTML and CSS for front-end interaction, while Streamlit handles back-end processing efficiently. As illustrated in Fig 10, the web application successfully loaded the saved model and, upon predicting the class for the provided image, indicated a classification of “Normal” with a confidence score of 100%.

### Comparison with other study

Table 13 presents a comparison of the performance of the current work with related studies on lung cancer detection and classification. A broad range of computer vision and deep learning networks has been employed in recent research. The proposed knowledge distillation technique developed in this research achieves comparable accuracy levels while significantly reducing model complexity and training time, making it suitable for resource-limited edge devices.

**Table 13:** Performance comparison of the proposed system with existing works.

Ref.	Dataset	Network	Metrics	# of parameters Time per epoch
[11]	NIH	EfficientNetB7	Accuracy: 96.1%	N/A
[15]	CT	CNN	Sensitivity: 0.97 Specificity: 0.99 AUC: 0.98	172.19s
[16]	LIDC-IDRI	VCNet	Accuracy: 99.49%	166k
[17]	NSCLC Radiomics	ViT	Dice coefficient: 0.7468 Hausdorff Distance: 15.336	N/A
[18]	Private data	Swin Transformer	Accuracy: 96.16%	N/A
[19]	LIDC-IDRI LUNA16	KD ConvNeXt	Accuracy: 85.64% F1: 0.7717	N/A
[20]	LUNA16	Enhanced Swin Transformer	Accuracy: 82.26%	88M
[21]	LUNA16	MAED	Sensitivity: 89.1%	N/A
[22]	LIDC-IDRI	Multi-scale MobileViT	Accuracy: 94.04% AUC: 0.9636	N/A
This work	NSCLC Radiomics	Knowledge Distillation Teacher: ViT TA: ResNet152v2 Student: CNN	Accuracy: 94.53% F1: 0.9465	147k (TA) 25k (Student) 5.09s (TA) 5.01s (Student)

### Limitations

This work implements a three-phase ViT-ResNet152v2-CNN (teacher-TA-student)-based explainable knowledge distillation technique for lung cancer classification employing the NSCLC-Radiomics dataset. The limitations of this study are briefly described below.

- This study exclusively utilizes the NSCLC-Radiomics dataset. Potential biases inherent in this dataset will negatively impact the performance of both the baseline and distilled learning models.
- This work employs a single-teacher model using the ViT transformer. Multi-teacher distillation techniques, such as ensemble and souping methods, have not yet been explored.
- The scalability of the applied models to larger and more diverse datasets with multiple data modalities has not been assessed.
- Furthermore, the black-and-white CT scan samples from the NSCLC-Radiomics dataset have not been combined with 3D and color or RGB images from various sources to create a more comprehensive database.

## Conclusions

In this work, a deep learning-based knowledge distillation has been developed with an intermediate model known as a teaching assistant to identify the different categories of NSCLC. An open-source extensive dataset NSCLC-Radiomics, containing 51,215 images of 422 patients and five distinct classes, has been used. We preprocessed the imbalanced dataset through several steps like resizing, labeling, cropping, applying normalization, etc. A cost-sensitive learning technique is applied to address the imbalanced problem of the employed dataset. Next, a wide range of deep learning and transfer learning frameworks are applied. The transformer-based ViT model is used as the teacher, and ResNet152v2 and a custom-built CNN are utilized as TA and student models, respectively. With optimized hyperparameters (alpha = 0.7 and temperature = 7), the TA and student networks obtain the highest accuracies of 90.99% and 94.53% accuracies, respectively. The primary objective of this study of reducing training time for memory-constraint edge devices is served using the applied distilled knowledge techniques. XAI with a partition explainer with a simple image has been developed to analyze the class’s importance by generating Shapley values. Finally, a user-friendly web application has been designed that inputs images from users to predict cancer types using the distilled student model. In the future, combining ensemble methods and amalgamating predictions derived from multiple models can be applied to knowledge distillation techniques. This approach is anticipated to yield more resilient predictions and enhance overall performance. Self-distillation and adopting a multistep process guided by insights obtained from prior outcomes can be initiated. 3D and color or RGB can be combined with CT scan black and white images to create a more comprehensive database. The applied models can be cross-validated with open-max system and domain adaptation techniques.
